# Mid-term Outcomes of Patella Resurfacing During Total Knee Arthroplasty (TKA): Clinical, Functional, and Radiographic Insights

**DOI:** 10.7759/cureus.75679

**Published:** 2024-12-13

**Authors:** Supreet Bajwa, Kunal Aneja, Ravi Teja Rudraraju, Ponnanna Machaiah, Haresh P Bhalodiya, Rakesh Kumar Singh, Vivdh Makwana, Avtar Singh, Vivek Logani, Buddhadev Chatterjee, Devendra Singh Solanki, Hemant Wakankar, Sanjeev Mahajan, Chandrashekhar Yadav, Ashok Kumar Thakkar, Udita Chandra, Sanaa Ansari, Shivadharshni Sivakumar

**Affiliations:** 1 Orthopaedics, Wockhardt Hospitals, Mumbai, IND; 2 Orthopaedics, Max Super Speciality Hospital, Delhi, IND; 3 Orthopaedics and Rehabilitation, Naveda Healthcare Centre, Delhi, IND; 4 Orthopaedics, Apollo Hospitals, Hyderabad, IND; 5 Orthopaedics, Sri Venkata Sai (SVS) Medical College, Mahbubnagar, IND; 6 Orthopaedics, Sparsh Hospital, Yeshwanthpur, Bengaluru, IND; 7 Orthopaedics, Saviour Hospital, Ahmedabad, IND; 8 Orthopaedics, Global Hospital for Joint Replacement, Kanpur, IND; 9 Orthopaedics, Navneet Hi-Tech Hospital, Mumbai, IND; 10 Orthopaedics, Amandeep Hospital, Amritsar, IND; 11 Joint Replacement and Sports Injury, Paras Hospital, Gurugram, IND; 12 Orthopaedics, Apollo Gleneagles Hospitals, Kolkata, IND; 13 Orthopaedics, Artemis Health Institute, Gurugram, IND; 14 Orthopaedics, Deenanath Mangeshkar Hospital and Research Centre, Pune, IND; 15 Orthopaedics, Fortis Hospital, Ludhiana, IND; 16 Joint Replacement Surgery, Primus Super Specialty Hospital, Delhi, IND; 17 Orthopaedics, All India Institute of Medical Sciences, New Delhi, Delhi, IND; 18 Clinical Research and Medical Writing, Meril Life Sciences Private Limited, Vapi, IND

**Keywords:** inflammatory arthritis, osteoarthritis, patella resurfacing, posterior stabilized knee, range of motion (rom), total knee system

## Abstract

Aim

The primary objective of the study was to evaluate the mid-term implant survivability, rate of revisions, and clinical and functional outcomes following patella resurfacing during total knee arthroplasty (TKA) utilizing posterior stabilized (PS) total knee system (TKS).

Methods

A prospective, single-arm, multi-center, post-marketing surveillance encompassed patients with end-stage primary knee osteoarthritis (OA) or inflammatory arthritis. The time points of the study included baseline, six weeks, six months, one year, and three years post-operatively. Clinical outcomes included Western Ontario and McMaster Universities Osteoarthritis Index (WOMAC) score, Short Form-36 questionnaire (SF-36), and Knee Society Score (KSS) for quality of life (QoL). Radiographs assessed loosening, patella tracking, and implant longevity. Functional outcomes were assessed by range of motion (ROM).

Result

The study included 74 patients undergoing patella resurfacing during TKA with a PS all-poly component TKS at 10 centers in India. Among the study population, 85% were female, and the average age of the population was 65.13±7.20 years. End-stage OA (70 patients) and inflammatory arthritis (four patients) were the prevalent conditions. Patella sizes used were: 25 mm (n=1), 28 mm (n=29), 31 mm (n=40), and 34 mm (n=4). Primary outcomes showed implant survival was 100% with no revisions after three years. Local soft tissue infections and discomfort affected 3.2%, with no additional adverse events. Radiographs showed well-implanted patellar components with no misalignment or wear after three years. Secondary outcomes showed a significant three-year increase in mean ROM from 85.50°±15.02° to 122.45°±2.44°. After three years, clinical and functional KSS improved to 90.36±3.72 (baseline: 21.11±14.49) p<0.001 and 97.95±3.67 (baseline: 27.16±13.22) p<0.001, respectively. WOMAC values for pain, stiffness, and difficulty decreased significantly (p<0.001) over the three-year duration. SF-36 evaluating QoL showed substantial improvements (physical functioning, role limitation, and general health).

Conclusion

The study highlights the success of patella resurfacing during TKA, demonstrating excellent implant survival, improved functional outcomes, and QoL over a three-year period.

## Introduction

Total knee arthroplasty (TKA) stands as a transformative intervention for patients with end-stage osteoarthritis (OA) or inflammatory arthritis, offering significant relief from pain and improvement in joint function [1]. However, the role of patella resurfacing during TKA remains a subject of ongoing debate within the orthopedic community [2]. The patellofemoral joint, particularly the patella, plays a crucial role in knee biomechanics, and addressing its pathology is essential for optimizing TKA outcomes [3].

Patella resurfacing involves the replacement of the patellar surface with an artificial implant, aiming to mitigate complications such as anterior knee pain, maltracking, and instability [4]. There is no unanimous consent over patellar resurfacing and no clear pathway or standard course of treatment quoted in the available literature. The best choice of treatment depends on the operating surgeon and the diseased condition, and it ranges from “routinely or selectively resurfacing” to “routinely not resurfacing.” Proponents of patellar resurfacing highlight the benefits of the technique as it helps reduce pain, improves patient satisfaction post-surgery, and decreases rates of reoperations [2,5,6]. They assert that this procedure contributes to enhanced patellar stability, reduced pain, and improved functional outcomes. On the contrary, critics express concerns about potential complications, including patellar fracture, clunk syndrome, and increased wear on the implant [7,8].

The research investigating the efficacy and long-term outcomes of patella resurfacing during TKA has increased in recent years [9-11]. Despite the growing evidence, controversies persist regarding the indications, patient selection criteria, and potential risks associated with patella resurfacing.

The aim of the current study was to understand and assess the functional outcome of posterior stabilized (PS) total knee system (TKS) with patella resurfacing during TKA in patients suffering from OA and inflammatory arthritis. The study also aimed to observe the overall implant survival over a period of three years through radiographic assessment, while also noting the clinical and functional outcomes in this cohort.

## Materials and methods

Population

This is a prospective, multicenter, single-arm, real-world assessment of patients suffering from end-stage OA/inflammatory arthritis undergoing primary TKA needing patellar resurfacing. Inclusion criteriaincluded: male or non-pregnant female patients aged 18 years or older at the time of the study; patients willing and able to provide written informed consent by signing and dating the Institutional Review Board or Ethical Committee-approved informed consent form; patients requiring TKA; and patients willing and able to comply with post-operative scheduled clinical and radiographic evaluations. Exclusion criteria included: patients with body mass index (BMI) ≥40kg/m^2^; patients with an active infection within the affected knee joint; patients with a neuromuscular or neurosensory deficiency that may limit the ability of the patient to evaluate the safety and efficacy of the device; patients with a known allergy to metals; patients unwilling or unable to sign the informed consent form; patients with short life expectancy less than five years (e.g., cancer, HIV/AIDS); patients with a history of deep vein thrombosis or other thrombotic disorders; and patients with conditions such as parkinsonism, locomotors ataxia, spinal scoliosis or kyphosis, and stroke that would affect their locomotors stability and interfere with assessment of ROM of the target knee.

The study was approved by the local review board of the respective sites and informed written consent was obtained from the patients pre-operatively. All patients were followed up for three years post-operation with time intervals of six weeks, six months, one year, and three years.

Endpoints

Demographic data including age, gender, and medical comorbidities were noted in the patient’s database and medical records. Mechanical and anatomical axes and mechanical axis deviation were radiographically assessed in pre-operative radiographs. Complications were assessed through standardized telephone interviews and clinical records. The primary outcomes included implant survivorship, wear, and osteolysis, examined using post-operative follow-up X-rays. The secondary outcomes were assessed using the Knee Society Score (KSS) both clinical and functional, the Short Form 36 questionnaire (SF-36), and the Western Ontario and McMaster Universities Osteoarthritis Index (WOMAC) at six weeks, six months, one year, and three years.

Methods

This study encompassed multiple surgeons in the evaluation and treatment of eligible patients. The indication for surgery and selection of the surgical technique was made by the attending surgeon. The patients were operated on using the standard medial parapatellar approach, and the PS Freedom® Total Knee System (Maxx Orthopedics Inc., Philadelphia, USA) was implanted in all patients. In all instances, the patella resurfacing was performed. Tourniquets were employed in all surgical procedures as an integral component of the technique. Additionally, each patient was offered intravenous prophylactic antibiotics prior to the surgery, followed by three doses of antibiotics after the operation.

Full weight-bearing mobilization as tolerated with a walker or crutch and stair climbing was recommended to all patients post-operatively till three months. Physiotherapy for three months with routine medical examinations was suggested to all patients during discharge. The healthcare regimen incorporated the utilization of acetaminophen and non-steroidal anti-inflammatory drugs (ketoprofen, ketorolac, or diclofenac) for analgesic management. For 21 days (prophylactic period), a continuous thromboprophylaxis regimen (rivaroxaban, orally) was recommended to all enrolled patients.

Statistical assessment

The findings were presented as the mean ± standard deviation for continuous parameters and as the count (percentage) for categorical variables. Continuous variables that exhibited a normal distribution were subjected to paired t-tests for dependent samples, while the Wilcoxon signed-rank test was applied when a normal distribution could not be assumed. Statistical significance was defined as p<0.05. Statistical analysis was performed using the RStudio software (Posit PBC, Boston, USA). 

## Results

The study enrolled 74 patients who underwent patellar resurfacing during TKA with PS implant across 10 centers in India. The average age of the enrolled patients was 65.1±7.2 years with female patients dominating the study (n=63 (85%)). The average BMI was 26.2±4.2 kg/m^2^ with end-stage OA (n=70) and inflammatory arthritis (n=4). The comorbidities observed were hypertension (58.6%), diabetes mellitus 29.3%, hypothyroidism 20.7%, dyslipidemia (10.4%), and previous joint surgery was observed in 8.6% of the cohort. The baseline and medical history of patients are described in Table 1.

**Table 1:** Baseline characteristics and medical history of patients. BMI: Body mass index; bpm: Beats per minute; PSVT: Paroxysmal supraventricular tachycardia

Variables	Patients n=74
Age, years, mean ± SD	65.1±7.2
Gender, n (%)	
Male	11 (15)
Female	63 (85)
BMI, kg/m^2^, mean ± SD	26.2±4.2
Heart rate, bpm, mean ± SD	78.6±10
Systolic blood pressure, mmHg, mean ± SD	133.8±13.1
Diastolic blood pressure, mmHg, mean ± SD	80.9±7
Primary diagnosis, n (%)	
Osteoarthritis	70 (94.6)
Rheumatoid arthritis	4 (5.4)
Medical history, n (%)	
Diabetes mellitus	22 (29.3)
Previous joint surgery	6 (8.6)
Dyslipidemia	8 (10.4)
Hypertension	43 (58.6)
Ischemic heart disease	1 (1.7)
History of other illnesses, n (%)	
Hypothyroidism	15 (20.7)
Hyperuricemia	1 (1.7)
Migraine	1 (1.7)
Obstructive compulsive disorder	1 (1.7)
Prostatomegaly	1 (1.7)
PSVT	1 (1.7)

**Figure 1:**
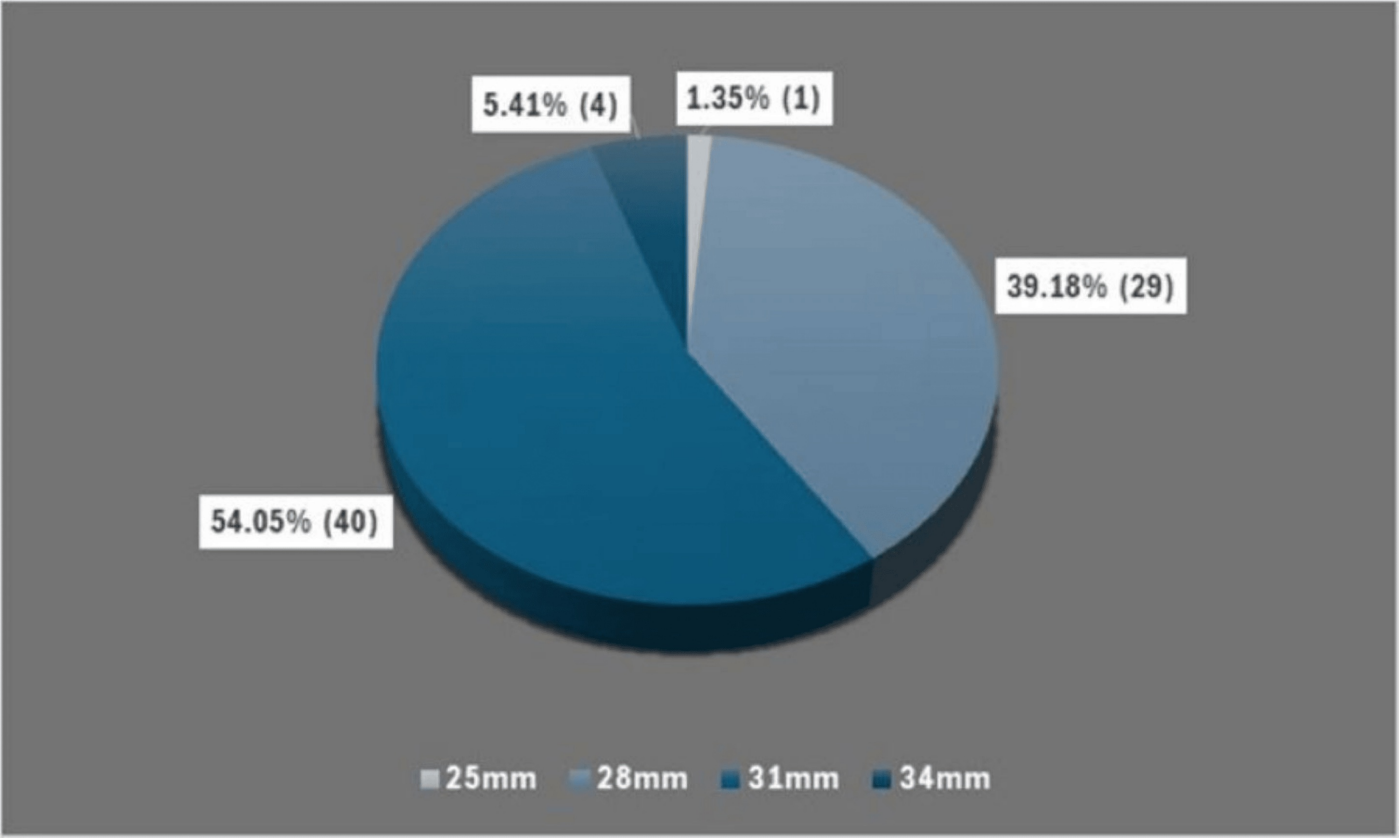
Details of total patellar component size used in study cohort.

A total of 74 implants of varying sizes, 25 mm (n=1), 28 mm (n=29), 31 mm (n=40), and 34 mm (n=4), were used (Figure 1).

In the cohort of 74 patients, each patient received a total of 37 left knee implants and an equal number of 37 right knee implants (Table 2).

**Table 2:** Implant details.

Parameters	
Type of implant	74 posterior stabilized implanted
Total knees implanted	
Left knees	37
Right knees	37

Primary outcomes

Implant survivorship and rate of revision were the primary endpoint of the study, which were observed to be 100% with an absence of revision in any of the patients over the period of three years (Table 3). There were no major complications during the follow-up period of three years. Superficial soft tissue infection was observed in 3.2% (n=2) patients which was managed with regular dressing and oral antibiotics. No organism was isolated in the culture swab among these patients, whereas one patient suffered from deep vein thrombosis and was medically managed.

**Table 3:** Primary outcomes showing 100% implant survivorship and absence of cumulative revision rate.

S.No.	Revision procedure	6 weeks	6 months	1 year	3 years
1	Femoral implant	0	0	0	0
2	Tibial implant	0	0	0	0
3	Patellar implant	0	0	0	0
4	All implant	0	0	0	0

**Figure 2:**
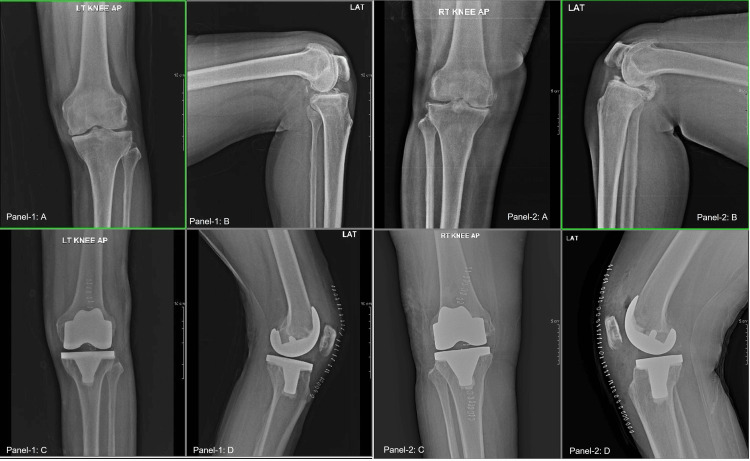
Panels 1A-B showing the AP and lateral view of the left diseased knees, respectively. Panels 1C-D showing the AP and lateral view of the implanted knee with patella resurfacing. Panels 2A-B showing the AP and lateral view of the right diseased knees, respectively. Panels 2C-D showing the AP and lateral view of the implanted knee with patella resurfacing, respectively. AP: Antero-posterior

Post-operative radiographs showed well-implanted patellar components (Figure 2). During the three years of follow-up, no loosening or wear was observed (Figure 2).

Secondary outcomes

**Figure 3:**
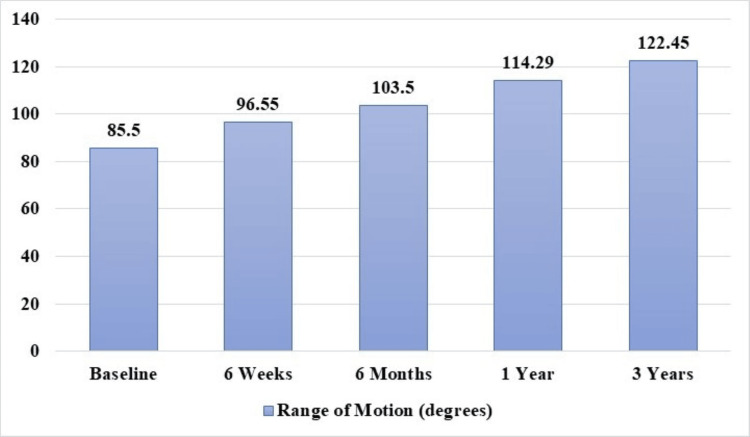
Increase in ROM scores over the course of three years compared to their pre-operative (baseline) scores, p<0.001. ROM: Range of motion

At the three-year follow-up, it was noted that the mean ROM in patients significantly improved to 122.5°±2.4° from a mean pre-operative score of 85.5°±15°, p<0.001 (Figure 3).

**Figure 4:**
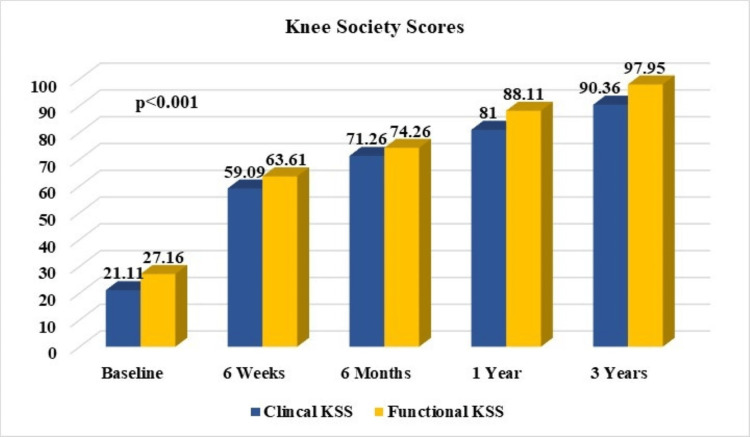
KSS (clinical and functional) of three-year follow-up period in comparison to their pre-operative (baseline) scores. KSS: Knee Society Score

The initial average clinical KSS before surgery was found to be lower (21.1±14.5), but it showed a considerable improvement to 71.3±13.1 after six months (p<0.001). Furthermore, there was a continued improvement detected at the three-year mark, with an average score of 90.4±3.7 (p<0.001). A similar trend was noted in the functional KSS, with a pre-operative score of 27.2±13.2 increasing to 97.9±3.7 at the three-year mark, (p<0.001) (Figure 4).

A total of 94% of the patients expressed satisfaction post-TKA, and at the three-year mark, 96% of the patients indicated satisfaction with enhanced functional activity and decreased discomfort.

**Figure 5:**
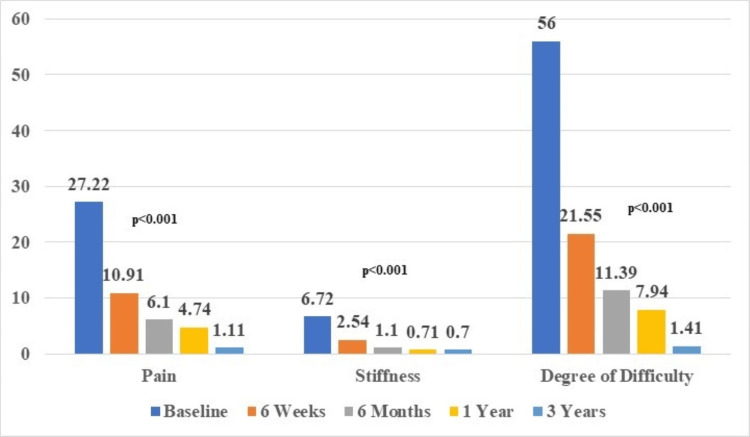
WOMAC scores of three-year follow-up period in comparison to their pre-operative (baseline) scores. WOMAC: Western Ontario and McMaster Universities Osteoarthritis Index

The post-operative assessment of pain, stiffness, and difficulty using the WOMAC scoring system showed a decrease in all three parameters for the whole three-year research period. The data presented in Figure 5 illustrate the initial high scores for pain (27.2±3.1), stiffness (6.7±0.9), and degree of difficulty (56±6.1), which subsequently decreased to 1.1±1.7, 0.7±0.6, and 1.4±3.8 at three years, respectively (p<0.001).

SF-36 with higher scores indicates better physical functioning, fewer physical health-related limitations, and a more positive perception of general health. The assessment of quality of life (QoL) using the SF-36 indicated a significant reduction in pain and an improvement in overall health and functionality, both physical and emotional, following TKA with patellar resurfacing using PS knees. As shown in Table 4, the baseline scores for physical functioning (7.8±10.7), role limitations due to physical health (0.00±0.00), and general health (43.6±21.8) showed a marked and continuous increase after surgery. Over the course of three years, these scores continued to improve 87.5±15.6, 62.05±23.4, and 78.3±16.7, respectively. Table 4 provides a detailed presentation of these findings.

**Table 4:** SF-36 assessing the quality of life of patients at various timepoints. SF-36: Short Form-36 questionnaire

Variables	Baseline	6 weeks	6 months	1 year	3 years	p-value (baseline vs. 3 years)
Physical functioning	7.84±10.68	49.91±25.81	66.75±21.66	70.44±16.64	87.50±15.64	<0.001
Role limitations						
- Due to physical health	0.00±0.00	62.07±45.94	81.14±35.13	92.98±21.00	62.05±23.35	<0.001
- Due to emotional problems	30.46±44.71	68.97±42.74	81.87±34.54	90.64±28.00	73.21±21.48	<0.001
Energy/fatigue	38.97±18.03	53.10±12.56	56.23±11.74	58.77±11.07	55.71±11.18	<0.001
Emotional well-being	46.69±20.01	67.24±11.25	69.82±7.97	72.70±7.71	62.57±12.24	<0.001
Social functioning	37.28±20.48	60.99±15.19	66.01±12.89	71.93±12.11	80.58±11.66	<0.001
Pain	22.72±14.18	54.14±14.60	66.01±15.22	72.89±12.35	78.30±16.69	<0.001
General health	43.62±21.80	64.74±14.85	67.28±11.99	70.88±9.73	82.68±9.77	<0.001
Health change	20.26±16.53	79.31±9.53	87.72±12.61	90.79±12.17	96.43±11.11	<0.001

## Discussion

We have assessed and reported the mid-term clinical and functional outcomes following primary TKA with patella resurfacing within the Indian population, utilizing PS TKS. In this cohort, no revision surgeries were required, resulting in a 100% implant survivorship rate at the three-year follow-up. The functional outcomes observed in our study were favorable and consistent with those reported in the existing literature.

As documented by Mishra et al. (2021) in a prospective observational study spanning two years, no complications were reported in the cohort of patients who underwent patellar resurfacing (n=20), while one patient in the non-resurfacing group (n=20) experienced intraoperative patellar maltracking [12]. Our study, which included a cohort of 74 patients, demonstrated an absence of intraoperative patellar maltracking and other complications over a three-year follow-up period. These outcomes reinforce the safety and effectiveness of patellar resurfacing in TKA, further validating its role in improving surgical outcomes.

The primary results of this study demonstrated good outcomes with no radiographic evidence of implant wear or loosening and anterior knee pain at three years follow-up. Furthermore, the results from assessments using the KSS, ROM, SF-36, and WOMAC scores showed a statistically significant difference between the pre-operative and post-operative evaluations over the three-year follow-up period (p<0.001). The significance of acknowledging that procedures were conducted by different surgeons across multiple centers lies in the recognition of the study's real-world applicability and generalizability. This variability in surgical approach reflects the diverse practices encountered in clinical settings, allowing for a more comprehensive assessment of the safety and effectiveness of the PS knees. Additionally, the study provides insights into the adaptability of the implant and the ease of use for surgeons, thereby contributing to a more robust understanding of the practical implications of the study findings.

Previous studies have found that anterior knee pain typically becomes apparent during the initial phase following surgery, with a preponderance observed within the first 18 months [13,14]. An association has been observed between the rise in TKA survival rates and the simultaneous escalation in complications, including anterior knee pain, impingement, and damage to the patellar articular surface. Therefore, the decision to conduct long-term follow-ups to observe patients and gain insights into the implant's functionality, patient satisfaction, safety, and QoL improvement following patella resurfacing during primary TKA was consistent with existing literature. Furthermore, our study cohort did not experience any early or late complaints of anterior knee pain.

On the other hand, individuals who have patellar resurfacing may encounter diminished chances of survival because of various problems, including wear, loosening of the implant, fractures, patellar osteonecrosis, heightened risk of infection, and patellar subluxation. Based on the extant body of literature, it has been demonstrated that challenges associated with patellar resurfacing in TKA can be categorized into four main categories: encompassing patient-related aspects, issues pertaining to prosthetic design, surgical techniques, and material quality. The utilization of patella resurfacing has been observed to result in a decreased incidence of revision [15]. Our study concords with the report as we found that with patellar resurfacing utilizing PS knees, patients reported reduced pain and absence of any complications such as wound site infections, osteonecrosis, patellar subluxation, or requiring any revision.

In a randomized controlled study comparing the outcomes of patellar replacement with patellar retention, the defined outcomes included reoperations due to patellar issues, anterior knee pain, knee ratings, stair-climbing ability, and patient satisfaction [16]. The study's findings indicated that the resurfaced patella performed better than the non-resurfaced patella. Notably, the relative risk of requiring reoperation, experiencing significant anterior knee pain, and having pain during stair climbing was higher in the non-resurfaced group compared to the resurfaced group [16]. Similarly, in our cohort, patellar resurfacing was associated with significant pain relief, enhanced QoL, and improved functional outcomes. However, while the randomized controlled study emphasized the reduction in specific complications such as reoperations and anterior knee pain, our findings focused more broadly on the overall clinical and functional improvements. Both studies underscore the benefits of patellar resurfacing in TKA. Table 5 presents a comparative analysis of the results obtained in this investigation and those reported in the existing literature.

**Table 5:** Comparative analysis of the results obtained in this investigation and those reported in the existing literature. KSS: Knee Society Score; WOMAC: Western Ontario and McMaster Universities Osteoarthritis Index; ROM: Range of motion; SF-36: Short Form-36 questionnaire; TKA: Total knee arthroplasty; OKS: Oxford Knee Score; PROMs: Patient-reported outcome measures; KOOS: Knee Injury and Osteoarthritis Outcome Score; VAS: Visual analog scale

Authors	Procedural details	Outcomes	References
This study	Prospective, multi-center study	No revision, minor complications, improved KSS, WOMAC, SF-36. Increased ROM (122.45°±2.44°) during three years follow-up.	
Grela et al.	Systematic review and meta-analysis	Reduced anterior knee pain with patella resurfacing (95% CI = 0.44-0.96). No significant differences in PROMs. Resurfacing lowers the risk of revision surgery (RR = 0.63, CI = 0.42-0.94) and complications (RR = 0.54, CI = 0.39-0.74). Observational evidence suggests selective resurfacing increases revision risk (RR = 1.14, CI = 1.05-1.22) compared with resurfacing. Selective resurfacing is associated with higher pain risk (RR = 1.25, CI = 1.04-1.50) but lower revision risk compared with no resurfacing (RR = 0.92, CI = 0.85-0.99).	[10]
Simpson et al.	Systematic review	Patellar resurfacing alongside a non-friendly TKA implant was linked with significantly improved OKS and reduced reoperation rates. The importance of considering implant design should be recognized when contemplating patellar resurfacing.	[17]
Aunan et al.	Single-center, randomized, double-blind study	Statistically significant improvements in the mean sub-scores for KOOS were observed post-surgery favoring patellar resurfacing, particularly in sport/recreation, knee-related quality of life, pain, and symptoms. However, no statistically significant differences were noted between the groups in the Knee Society Clinical Rating System, OKS, or VAS for patient satisfaction.	[5]
Gogia et al.	Prospective comparative study	The patellar resurfacing group demonstrated statistically significant enhancement compared to the non-resurfacing group in KSS clinical and functional scores, as well as the VAS, after one year.	[18]
Tang et al.	Review: grading of recommendations assessment, development, and evaluation (GRADE) framework	There were notable decreases in the patellar revision rate (RR 0.41, 95% CI (0.19, 0.88); P = 0.02). Patellar resurfacing substantially decreased the incidence of anterior knee pain and significantly lowered the occurrence of patellar clunk (RR 0.58, 95% CI (0.38, 0.88); P = 0.01).	[19]

The study underscores the importance of assessing patient-reported outcomes, as we observed excellent overall KSS, WOMAC, and SF-36 outcomes for our patients. Notably, patients who scored poorly pre-operatively reported higher rates of satisfaction level with the absence of complications during three-year follow-up.

In the context of TKA in India, there is a notable scarcity of data specifically addressing the outcomes of patellar resurfacing. Despite the increasing prevalence of TKA in the Indian population, most studies have focused on Western populations, leaving a significant gap in understanding the procedure’s efficacy and safety within an Indian context. This prospective study comprehensively evaluates the three-year clinical and functional outcomes of primary TKA with patellar resurfacing using a PS all-poly implant in Indian patients.

Given the unique demographic and lifestyle factors prevalent in India, such as a higher incidence of squatting and sitting cross-legged, understanding the impact of patellar resurfacing in this population is particularly important.

Limitations

It is essential to acknowledge the limitations inherent in the research: involving a small group of patients and not taking into consideration other factors such as body mass index and comorbidities, which can be the contributing factors for long-term (five-year) failures leading to revision. Nonetheless, it contributes valuable insights into PS patellar implants and their survivability, as well as patient satisfaction in the functional evaluation of the Indian cohort who have undergone primary TKA with patellar resurfacing.

## Conclusions

The current investigation, involving the cohort of 74 individuals, provides compelling evidence about the efficacy of patella resurfacing utilizing PS implants during TKA. This success has been observed across various centers in India, specifically in the context of treating end-stage OA. The study demonstrates reliability, safety, and effectiveness of patella resurfacing as evidenced by a 100% implant survivorship rate over a period of three years, minimal complications, significant enhancements in ROM, and positive patient-reported outcomes, all without any instances of revision. The findings suggest that the patella resurfacing during TKA may serve as a viable therapeutic approach utilizing PS implants. This could potentially enhance knee functionality, mitigate complications, and yield improvements in quality of life, particularly when employing the latest implant with varied sizes, tailored to individual patient requirements.
